# A Rare Hybrid Presentation: Coexistence of Necrotizing and Histiocytoid Variants of Sweet Syndrome (SS) in a Patient With Acute Myeloid Leukemia (AML)

**DOI:** 10.7759/cureus.105085

**Published:** 2026-03-12

**Authors:** Aya Al Rawahi, Zamzam Al-Qutaiti, Maimouna Alfarsi

**Affiliations:** 1 Dermatology, Ministry of Health Holdings, Muscat, OMN; 2 Dermatology, Al Nahdha Hospital, Muscat, OMN

**Keywords:** acute myeloid leukemia (aml), hematology-oncology, lingual hematoma, necrotizing fascitis, necrotizing sweet syndrome, sweet's syndrome

## Abstract

Sweet syndrome (SS) is a rare, acute, febrile neutrophilic dermatosis marked by neutrophilic infiltration into the skin, blood, and various organ systems. It manifests as painful skin eruptions, which can range from violaceous papules and plaques to vesicles and ulcers, accompanied by peripheral blood neutrophilia and fever. Sweet syndrome has three subtypes: classical, drug-induced, and malignancy-associated. There are different clinical and pathological variants of SS. However, rare variants, such as necrotizing SS and histiocytoid SS, can complicate the clinical and pathological picture, particularly in the context of hematologic malignancies.

We report a case of a female patient in her late 50s with acute myeloid leukemia (AML) who developed rapidly progressing, painful, necrotic skin lesions. Clinically, the presentation raised immediate suspicion for necrotizing fasciitis. However, histopathological examination revealed a dense dermal infiltrate of mononuclear cells resembling histiocytes. Immunohistochemical staining confirmed these cells were immature myeloid precursors (MPO+/CD68+), consistent with histiocytoid SS. Despite the clinical appearance of full-thickness necrosis, the patient responded dramatically to high-dose systemic corticosteroids, avoiding the need for extensive surgical debridement.

This case highlights the rare overlap of necrotizing and histiocytoid variants of SS as a paraneoplastic phenomenon in AML. It underscores the importance of recognizing these atypical presentations to prevent misdiagnosis of infection and to ensure the timely initiation of immunosuppressive therapy. Clinicians should remain vigilant, as these variants may serve as a sentinel marker for underlying or relapsed leukemia.

## Introduction

Sweet syndrome (SS) typically presents with painful erythematous papules, plaques, and fever. The histological features of SS are characterized by a diffuse neutrophilic infiltrate with dermal papillary edema that can involve the mid-dermis to the subcutis [1]. Although the exact pathogenesis is yet to be fully elucidated, it is thought to be from a synergistic interaction between genetic susceptibility and exogenous environmental factors [2]. SS has three main subtypes: classical, drug-induced, and malignancy-associated. Approximately 15-20% of SS cases are malignancy-associated, with acute myeloid leukaemia (AML) being the most common underlying hematologic trigger. In such cases, the disease may present atypically or with increased severity [3].

In recent years, a broader spectrum of SS presentations has been recognized, with several reports highlighting novel clinical and histological variants. These atypical variants have broadened the clinical scope of SS, moving it beyond the classical presentation and introducing distinct complexities in both identification and treatment [4].

The clinical variants of SS include bullous SS, cellulitis-like SS, neutrophilic dermatosis of the dorsal hands, and necrotizing SS (NSS). The pathological variants of SS are classified according to the dominant type of inflammatory cell infiltrate. For example, histiocytoid, eosinophilic, lymphocytic, normolipemic xanthomized, and cryptococcoid [4]. Overlap between different variants has been noted in the literature. 

Clinically, NSS represents a severe, locally aggressive variant of SS that mimics necrotizing fasciitis. Its presentation with fever, systemic inflammatory response, and deep-tissue necrosis often leads to initial misdiagnosis as necrotizing fasciitis. The association of NSS and AML can easily be mistaken for leukemic or infectious skin involvement, which can potentially delay appropriate treatment [5].

The pathological variant of histiocytoid SS (HSS) is characterised by a dermal infiltrate of immature neutrophils that resemble histiocytes, with immunohistochemical stain (IHC) positive for CD68 and myeloperoxidase (MPO).

## Case presentation

A 58-year-old female with a history of hypertension, diabetes mellitus, dyslipidemia, hypothyroidism, and advanced AML on palliative care was admitted with a 1-week history of erythematous, painful skin eruption over the limbs, mostly the right forearm. It started as painful papules, which enlarged into plaques and deep swelling. It was associated with persistent episodes of fever of 38 °C.

**Figure 1:**
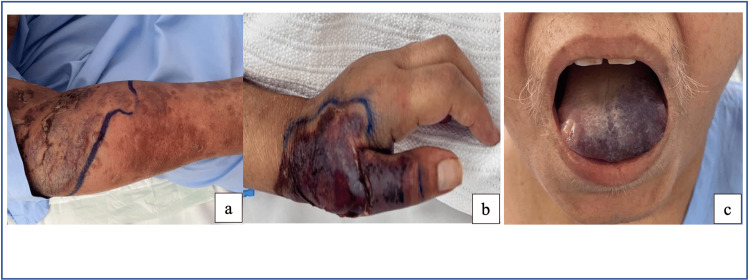
(a) Deep erythematous swelling over the right forearm, with overlying tense blisters. (b) Violaceous patch with necrosis over the dorsum of the left hand. (c) Violaceous plaque over the anterior aspect of the tongue.

On clinical examination, there were multiple red to violaceous papules and plaques with underlying swelling on the right forearm (Figure 1a). The initial workup diagnosis by the medical team was cellulitis/necrotizing fasciitis. She was treated with broad-spectrum IV antibiotics for three weeks. Despite antibiotic therapy, the patient's condition failed to improve; instead, it deteriorated, with the lesions progressing to tissue necrosis. In addition, a new necrotic and hemorrhagic bulla developed over the right thumb after the placement of an IV cannula (Figure 1b). Simultaneously, the patient developed a sudden onset of painful, violaceous swelling on the anterior aspect of the tongue (Figure 1c), but unfortunately, she refused biopsy of this site.

**Figure 2:**
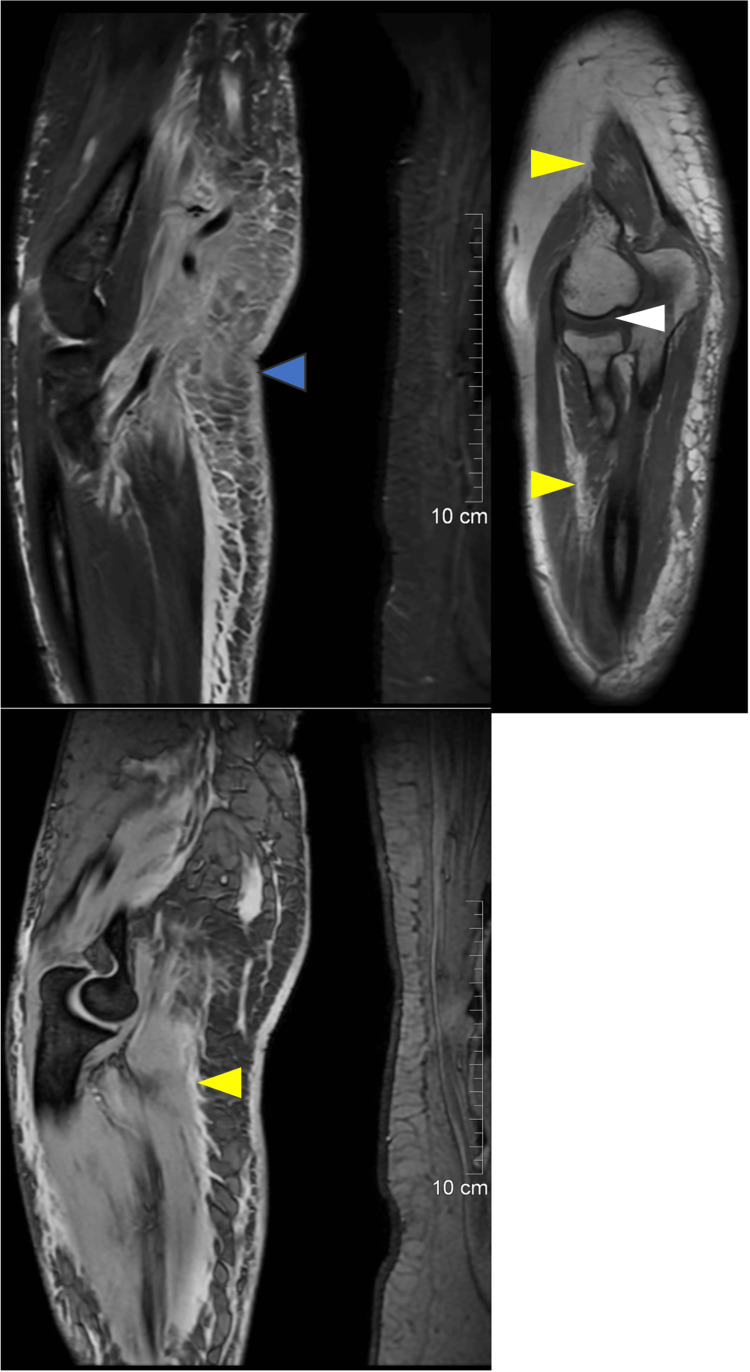
MRI elbow There is diffuse subcutaneous oedema and post-contrast enhancement with associated diffuse, almost circumferential, skin thickening (blue arrow). There is intramuscular edema and postcontrast enhancement of the anterior compartment muscle, mainly involving the distal portion of the brachialis and biceps muscles (yellow arrows). In addition, there is intramedullary bone marrow edema of the distal metadiaphysis of the humerus and proximal metadiaphysis of both the ulna and radius with post-contrast enhancement (white arrow).

An MRI of the right forearm was done (Figure 2) to assess for necrotizing fasciitis. Features were suggestive of diffuse cellulitis involving the distal arm and forearm. There was no drainable, organized fluid collection. Findings equivocal for osteomyelitis involved the distal humerus and radius metadiaphysis. Wound and blood cultures were negative. Her antibiotics were upgraded from intravenous tazocin to meropenem and clindamycin, but clinically, she did not respond.

**Figure 3:**
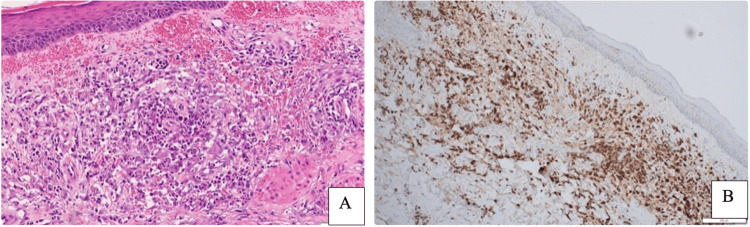
(A) 20X: Red blood cells (RBC) extravasation in the upper dermis, with numerous neutrophilic and mixed inflammatory infiltrates. (B) 20X: Positive methyelproxidase stain (MPO) confirming the presence of neutrophilic precursors, histiocytoid cells.

Our team was consulted, as the patient had failed multiple lines of antibiotics, with persistence of skin and oral mucosal eruptions. In light of the recurrent painful and necrotic plaques, swelling, hematoma, and fever, a skin biopsy was taken from the right forearm. The histopathology report showed the epidermis with mild hyperkeratosis and mild spongiosis. The upper dermis exhibited moderate neutrophilic infiltrate, numerous histiocytoid cells, and marked leukocytoclasia (Figure 3a). MPO stain, which is primarily used to identify cells of the myeloid lineage and is a significant tool to diagnose the histiocytoid variant of Sweet syndrome, was diffusely positive (Figure 3b). Thus, the histopathology was consistent with "histiocytoid Sweet syndrome. The final diagnosis was established clinically as NSS with pathological features of HSS.

She was managed with high doses of systemic corticosteroids: intravenous hydrocortisone 200 mg three times a day for 3 days, then twice a day for 5 days, followed by oral prednisolone (1 mg/kg) for 1 month, with gradual tapering.

**Figure 4:**
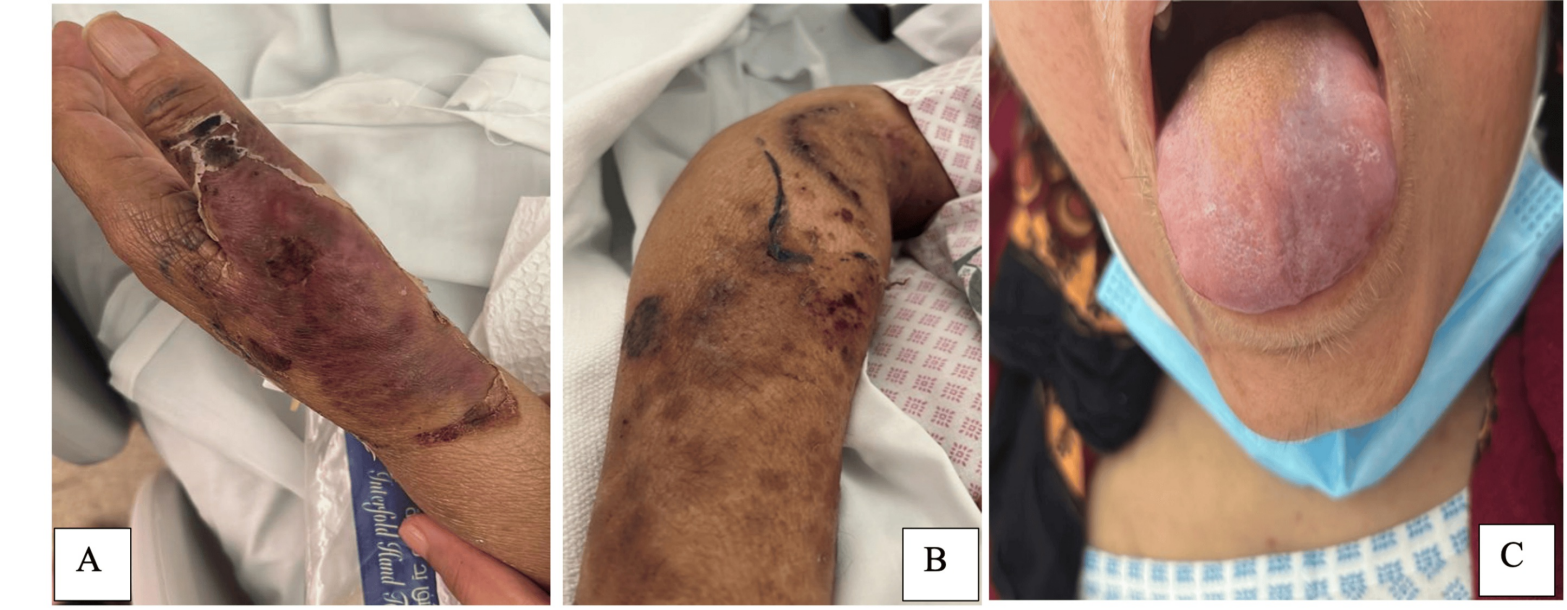
(a-c) Improvement in the lesions after starting IV hydrocortisone

The skin lesions resolved with post-inflammatory hyperpigmentation but no scarring (Figure 4). Surprisingly, the lingual hematoma healed as well with the initiation of steroids. Two months later, upon follow-up, there was no recurrence of any skin or mucosal lesions of SS.

The patient died of complications related to her underlying malignancy several months later.

## Discussion

NSS is an aggressive variant that histopathologically and clinically mimics necrotizing fasciitis [5,6]. However, differentiating between the two is crucial, as management strategies differ significantly. While both present with rapidly progressive necrosis and systemic inflammation, NSS often exhibits the "pathergy phenomenon," where minor trauma or surgical debridement triggers further lesion exacerbation, as was described in our patient after introducing an intravenous cannula [7]. In our case, the patient progressed rapidly and developed hemorrhagic bullae, which quickly turned into necrotic plaques.

The rapid clinical presentation and acute onset of NSS closely resemble those of necrotizing fasciitis. Differentiating between the two conditions is crucial, as their treatments are fundamentally different. NSS is characterized by repeated negative wound cultures and clinical worsening despite surgical debridement and antibiotics, but it responds rapidly to corticosteroids [8,9]. As was noted in this case, the repeated wound and blood cultures remain negative. In contrast, necrotizing fasciitis occurs in the setting of an infectious process, with surgical debridement being the first-line treatment [9].

Histopathological evaluation remains the definitive diagnostic gold standard for NSS. The pathognomonic findings include a dense, diffuse neutrophilic dermatosis accompanied by marked papillary dermal edema, specifically characterized by the absence of primary vasculitis and a lack of demonstrable microbial pathogens [10]. In our case, the skin biopsy did not reveal typical neutrophil-dominated inflammation but rather a histiocytoid (mononuclear) infiltrate, leading to a diagnosis of the histiocytoid variant of Sweet syndrome, which was further confirmed with CD-68 and myeloperoxidase (MPO) stains.

In 2005, Requena et al. identified HSS as a unique subtype characterized by dermal swelling and immature myeloid cells. Histologically, it consists of superficial and deep dermal infiltrate of mononuclear cells, with a large, eccentric kidney‑shaped nucleus, conspicuous nucleolus, and eosinophilic cytoplasm. While these cells look like histiocytes, staining for CD-68 and MPO proved they are actually neutrophil precursors, distinguishing HSS from the standard neutrophil-heavy presentation of classic SS. Multiple cases of patients with myelodysplastic syndromes (MDS) being diagnosed with HSS have been reported. In comparison to classic SS, HSS demonstrates a higher correlation with hematological malignancies and MDS, whereas lymphoid malignancies appear to be less prevalent in HSS cases [11].

In their study of 13 HSS cases, Magro et al. observed a male predominance (8 men, 5 women) with an average age of 60. Over half the cohort (seven patients) suffered from myeloproliferative disorders, specifically MDS, in five instances. Beyond one patient with no comorbidities, others presented with conditions such as familial Mediterranean fever, various cancers, or associated triggers like COX-2 inhibitors and bortezomib therapy [12].

The coexistence of NSS and HSS was documented only once in a case report of a 66-year-old man who was diagnosed with chronic myelomonocytic leukemia, which transformed to AML and was treated with azacitidine therapy. He developed NSS with histopathological findings characteristic of the histiocytoid variant of SS [13].

In this case, our patient developed a unilateral lingual hematoma at the same time as SS. Surprisingly, the lingual hematoma and SS improved simultaneously after starting systemic steroids. However, there is no association between the two conditions reported in the literature, and this finding could be coincidental, as the biopsy of the tongue was not done to confirm the diagnosis. On the other hand, a lingual hematoma is more commonly associated with underlying hematological malignancies due to spontaneous bleeding and pancytopenia, while macroglossia was reported twice in association with SS [14].

## Conclusions

In conclusion, this case illustrates a rare and aggressive overlap of necrotizing and histiocytoid variants of Sweet syndrome (SS) in the setting of acute myeloid leukemia (AML). While the necrotizing clinical presentation often mimics life-threatening necrotizing fasciitis, the presence of a histiocytoid infiltrate on biopsy serves as a critical diagnostic clue toward a paraneoplastic etiology rather than an infectious one.

Recognizing these atypical variants is essential to avoid unnecessary surgical debridement and to initiate prompt corticosteroid therapy. This case reinforces the need for clinicians to maintain a high index of suspicion for neutrophilic dermatoses in hematologic patients, as these cutaneous manifestations may precede or signal the progression of the underlying malignancy.
